# Complex Fuzzy Set-Valued Complex Fuzzy Measures and Their Properties

**DOI:** 10.1155/2014/493703

**Published:** 2014-06-24

**Authors:** Shengquan Ma, Shenggang Li

**Affiliations:** ^1^College of Mathematics and Information Science, Shaanxi Normal University, Xi'an 710062, China; ^2^School of Information and Technology, Hainan Normal University, Haikou 571158, China

## Abstract

Let F*(*K*) be the set of all fuzzy complex numbers. In this paper some classical and measure-theoretical notions are extended to the case of complex fuzzy sets. They are fuzzy complex number-valued distance on F*(*K*), fuzzy complex number-valued measure on F*(*K*), and some related notions, such as null-additivity, pseudo-null-additivity, null-subtraction, pseudo-null-subtraction, autocontionuous from above, autocontionuous from below, and autocontinuity of the defined fuzzy complex number-valued measures. Properties of fuzzy complex number-valued measures are studied in detail.

## 1. Introduction

It is well known that additivity of a classical measure primly depicted measure problems under error-free condition. But when measure error was unavoidable, additivity could not fully depict the measure problems under certain condition. To overcome such difficulties, fuzzy measure has been developed. Research on fuzzy measures was very deep in those aspects: research based on a certain number of subsets of a classic set and a real value nonaddable measure (such as Choquet's content theory [1], Sugeno's measure theory [2]), research based on fuzzy sets and a real value measure (e.g., Zadeh's addable measure [3]), and especially the research on fuzzy value measures which generalizes the set value measure theory.

Being a newly developing theory developed in the later 1960s, set value measure had been applied in many fields [4–6]. After the appearance of fuzzy numbers, people naturally thought of related measure and integral. In 1986 Zhang [7] defined a kind of fuzzy set measure on *R* ^*n*^, in 1998 Wu et al. generalized the codomain of fuzzy measure to fuzzy real number field and defined the Sugeno integral of fuzzy number fuzzy measure [8], and Guo et al. also defined the *G* fuzzy value measure integral of fuzzy value function [9] which generalized the Sugeno integral about fuzzy value fuzzy measure to fuzzy set [10]. In 1989, Buckley presented the concept of fuzzy complex number [11] which inspired that people needed to consider the measure and integral problem about fuzzy complex number.

At the beginning of the 90s, Guang-Quan [12–21] introduced fuzzy real distance and discussed the fuzzy real measure based on fuzzy sets and then gave the fuzzy real value fuzzy integral and established fuzzy real valued measure theory on fuzzy set space. During 1991-1992, Buckley and Qu [22, 23] studied the problems of fuzzy complex analysis: fuzzy complex function differential and fuzzy complex function integral. During 1996–2001, Qiu et al. studied serially basic problems of fuzzy complex analysis theory, including the continuity of fuzzy complex numbers and fuzzy complex valued series [24], fuzzy complex valued functions and their differentiability [25], and fuzzy complex valued measure and fuzzy complex valued integral function [26, 27]. Wang and Li [28] gave the fuzzy complex valued measure based on the fuzzy complex number concept of Buckley, studied Lebesgue integral of fuzzy complex valued function, and obtained some important results.

As for applications of fuzzy complex number theory, Ramot et al. [29, 30] studied complex fuzzy sets and complex fuzzy logic, Dick [31] studied fuzzy complex logic more profoundly, Ha et al. [32] applied fuzzy complex set in statistical learning theory and obtained a key theorem of statistical learning theory, Fu and Shen [33] studied modeling problems of fuzzy complex number, and Fu and Shen [34] applied fuzzy complex in pattern recognition and classification and obtained important results. Please see [35–37] for other applications.

This paper will extend the classical measure to fuzzy complex number-valued measure, which can better express the interactions among the attributes (cf. [32, 34–37]) and, thus, is expected to have extensive applications in information fusion technology, classification technology, machine learning, pattern recognition, and other fields.  Section 2 is some preliminary notions (including fuzzy complex number, real fuzzy distance between two fuzzy real numbers, and two fuzzy complex numbers) and some basic operations and order relation of fuzzy complex.  Section 3 is prepared for the next section. We defined, based on Ha's work [38], the concepts of fuzzy complex distance and complex fuzzy set value complex fuzzy measure (an extension of fuzzy measure) on fuzzy complex number field. We also present, based on Zhang's work [21], the concepts of null-additivity, pseudo-null-additivity, null-subtraction, pseudo-null-subtraction, autocontinuous from above, autocontinuous from below, and autocontinuity of fuzzy complex value fuzzy complex measure on complex fuzzy number set (this measure has the properties PGP and SA/SB). In Section 4, we deduced some important properties on complex fuzzy set value complex fuzzy measure which are generalizations of the corresponding results in measure theory; we also obtain some results on related integral theory.

## 2. Preliminaries

### 2.1. Fuzzy Complex Numbers

In this paper, *R* is the set of all real numbers set, *K* is the set of all complex numbers, *X* is an ordinary set, F*(*R*) is the set of all real fuzzy numbers on *R*, Δ(*R*) is the set of all interval numbers, (*X*, *A*) is a measurable space (thus *A* is a *σ*-algbra), and F*(*K*) is the set of all fuzzy complex numbers on *K*. Let $F_{+}^{*}(R)=\{\tilde{a}:\tilde{a}\geq 0,\tilde{a}\in F^{*}(R)\}$ and let *F*_+_*(*K*) = $\left\{\tilde{A}+i\tilde{B}|\tilde{A},\tilde{B}\in F_{+}^{*}(R),i=\sqrt{-1}\right\}$.

**Definition 1 (see [12]).**

Let $\tilde{a},\tilde{b}\in F^{*}(R)$. Then the mapping $\left(\tilde{a},\tilde{b}):K\to [0,1\right]$ defined by $\left(\tilde{a},\tilde{b})(x+iy)=\tilde{a}(x)\wedge \tilde{b}(y\right)$ is called a fuzzy complex number, where $\tilde{a}$ is called the real part of $\left(\tilde{a},\tilde{b}\right)$ (written as Re$\left(\tilde{a},\tilde{b}\right)$) and $\tilde{b}$ is called the imaginary part (written as Im⁡(a~,b~)), and $i=\sqrt{-1}$. One will identify $\left(\tilde{a},\tilde{0}\right)$ with $\tilde{a}$ and, thus, think fuzzy complex numbers are an extension of fuzzy real numbers. The set of all fuzzy complex numbers on *K* is denoted by F*(*K*).

For any subsets *A*, *B* of *R*, write (*A*, *B*)≜*A* + *iB* = {*x* + *iy*∣*x* ∈ *A*, *y* ∈ *B*}. The operation ∘∈{+, −, ·} is described as follows:

1. *c*_1_′∘*c*_2_′ = (*Rec*_1_′∘*Rec*_2_′, *Im*⁡*c*_1_′∘*Im*⁡*c*_2_′) for any *c*_1_′, *c*_2_′ ∈ F*(*K*);
2. *c* · *c*_1_′ = (*a* *Rec*_1_′, *bIm*⁡*c*_1_′) for any *c*_1_′ ∈ F*(*K*) and *c* = (*a*, *b*) ∈ *K* ($c=a+ib,a,b\in R,i=\sqrt{-1}$).

*c*′ ∈ F*(*K*) is said to be a fuzzy infinity [21] (written as $\tilde{\infty }$) if one of the supports of a~=Rec′ and b~=Im⁡c′ is an unbound set. For any *c*_1_′, *c*_2_′ ∈ F*(*K*), one makes the following appointments:

* 
*c*_1_′ ≤ *c*_2_′* 
if and only if *Rec*_1_′ ≤ *Rec*_2_′ and *Im*⁡*c*_1_′ ≤ *Im*⁡*c*_2_′;
 
*c*_1_′ = *c*_2_′ if and only if *c*_1_′ ≤ *c*_2_′ and *c*_2_′ ≤ *c*_1_′;
 
*c*_1_′ < *c*_2_′ if and only if *c*_1_′ ≤ *c*_2_′ and *Rec*_1_′ ≤ *Rec*_2_′ or *Im*⁡*c*_1_′ < *Im*⁡*c*_2_′;
 
*c*′ ≥ 0 if and only if *Rec*′ ≥ 0, *Im*⁡*c*′ ≥ 0.

One uses A* to denote a family (which is obviously nonempty) of subsets of F*(*K*) that satisfies the following conditions:

1. for each $\tilde{A}\in A^{*}$, if $\tilde{B}=\{\inf{\tilde{A}}_{0}|{\tilde{A}}_{0}\subset \tilde{A}\}$ has upper bound, then $\sup\tilde{B}\in F^{*}\left(K\right)$;
2. for each $\tilde{A}\in A^{*}$, if $\tilde{C}=\{\sup{\tilde{A}}_{0}|{\tilde{A}}_{0}\subset \tilde{A}\}$ has lower bound, then $\sup\tilde{B}\in F^{*}\left(K\right)$.

### 2.2. Fuzzy Distance of Fuzzy Numbers

**Definition 2 (see cf. [21]).**

A mapping $\tilde{\rho }:F^{*}(R)\times F^{*}(R)\to F^{*}(R)$ satisfying the following conditions is called a fuzzy metric or a fuzzy distance on F*(*R*):

1. for any $\tilde{a},\tilde{b}\in F^{*}(R)$, $\tilde{\rho }(\tilde{a},\tilde{b})\geq 0$, and $\tilde{\rho }(\tilde{a},\tilde{b})=0$ if and only if $\tilde{a}=\tilde{b}$;
2. for any $\tilde{a},\tilde{b}\in F^{*}(R)$, $\tilde{\rho }(\tilde{a},\tilde{b})=\tilde{\rho }(\tilde{b},\tilde{a})$;
3. for any $\tilde{a},\tilde{b},\tilde{c}\in F^{*}(R)$, $\tilde{\rho }(\tilde{a},\tilde{b})\leq \tilde{\rho }(\tilde{a},\tilde{c})+\tilde{\rho }(\tilde{c},\tilde{b})$.

$\tilde{\rho }(\tilde{a},\tilde{b})$ is called the fuzzy distance of fuzzy real numbers $\tilde{a}$ and $\tilde{b}$.

**Example 3 .**

The mapping $\tilde{\rho }:F^{*}(R)\times F^{*}(R)\to F^{*}(R)$ defined by

(1) $$\begin{array}{c} \tilde{\rho }\left(\tilde{a},\tilde{b}\right)=\underset{\lambda \in \left[0,1\right]}{⋃}\lambda \left[\left|a_{1}^{-}-b_{1}^{-}\right|,\underset{\lambda \leq \eta \leq 1}{\sup}\left|a_{\eta }^{-}-b_{\eta }^{-}\right|\vee \left|a_{\eta }^{+}-b_{\eta }^{+}\right|\right] \end{array}$$

is a fuzzy distance on F*(*R*).

**Remark 4 .**

Analogously, a mapping $\tilde{\rho }:F^{*}(K)\times F^{*}(K)\to F^{*}(R)$ satisfying the following conditions is called a fuzzy metric or a fuzzy distance on F*(*K*):

1. for any $\tilde{a},\tilde{b}\in F^{*}(K)$, $\tilde{\rho }(\tilde{a},\tilde{b})\geq 0$, and $\tilde{\rho }(\tilde{a},\tilde{b})=0$ if and only if $\tilde{a}=\tilde{b}$;
2. for any $\tilde{a},\tilde{b}\in F^{*}(K)$, $\tilde{\rho }(\tilde{a},\tilde{b})=\tilde{\rho }(\tilde{b},\tilde{a})$;
3. for any $\tilde{a},\tilde{b},\tilde{c}\in F^{*}(K)$, $\tilde{\rho }(\tilde{a},\tilde{b})\leq \tilde{\rho }(\tilde{a},\tilde{c})+\tilde{\rho }(\tilde{c},\tilde{b})$.

$\tilde{\rho }(\tilde{a},\tilde{b})$ is called the fuzzy distance of fuzzy complex numbers $\tilde{a}$ and $\tilde{b}$.

**Example 5 .**

Let $\tilde{\rho }$ be a fuzzy distance on F*(*R*). Then the mapping *ρ*′ : F*(*K*) × F*(*K*) → F*(*R*) defined by

(2) ρ′(c1′,c2′)=ρ~(Rec1′,Rec2′)∨ρ~(Im⁡c1′,Im⁡c2′)

is a fuzzy distance on F*(*K*).

**Definition 6 (see cf. [12]).**

Let {*c*_*n*_′} ⊂ F*(*K*) and let *c*′ ∈ F*(*K*). {*c*_*n*_′} is said to converge to *c*′ according to a fuzzy metric *ρ*′ on F*(*K*) (written as lim⁡_*n*→*∞*_ *c*_*n*_′ = *c*′) if, for each *ε* > 0, there exists a positive integer *N* such that *ρ*′(*c*_*n*_′, *c*′) < *ε* for all *n* ≥ *N*.

## 3. Complex Fuzzy Set-Valued Complex Fuzzy Measures

The notion of complex fuzzy measure on family of classical sets was given in [26].

**Definition 7 (see [26]).**

Let $\overset{\wedge }{R^{+}}=[0,+\infty )$ and let $\overset{\wedge }{C^{+}}=\{x+iy|x,y\in \;\overset{\wedge }{R^{+}}\}$. A fuzzy measure on a *σ*-algebra *A* composed of subsets of *X* is a mapping $\mu :A\to \overset{\wedge }{C^{+}}$ which satisfies the following conditions:

1. *μ*(*ϕ*) = 0;
2. if *A* ⊂ *B*, then |*μ*(*A*)| ≤ |*μ*(*B*)|;
3. if {*A*_*n*_}_1_^*∞*^↑, then *μ*(⋃_*n*=1_^*∞*^ *A*_*n*_) = lim⁡_*n*→*∞*_ *μ*(*A*_*n*_);
4. if {*A*_*n*_}_1_^*∞*^↓ and |*μ*(*A*_*n*_0__)| < +*∞* for some *n*_0_, then *μ*(⋂_*n*=1_^*∞*^ *A*_*n*_) = lim⁡_*n*→*∞*_ *μ*(*A*_*n*_).

In this paper we need an expansion of this notion. First we defined the concept of fuzzy complex value distance.

**Definition 8 .**

A mapping $\tilde{\rho }:F^{*}(K)\times F^{*}(K)\to F^{*}(K)$ satisfying the following conditions is called a fuzzy complex value metric or a fuzzy complex value distance on F*(*K*):

1. for any $\tilde{a},\tilde{b}\in F^{*}(K)$, $\tilde{\rho }(\tilde{a},\tilde{b})\geq 0$, and $\tilde{\rho }(\tilde{a},\tilde{b})=0$ if and only if $\tilde{a}=\tilde{b}$;
2. for any $\tilde{a},\tilde{b}\in F^{*}(K)$, $\tilde{\rho }(\tilde{a},\tilde{b})=\tilde{\rho }(\tilde{b},\tilde{a})$;
3. for any $\tilde{a},\tilde{b},\tilde{c}\in F^{*}(K)$, $\tilde{\rho }(\tilde{a},\tilde{b})\leq \tilde{\rho }(\tilde{a},\tilde{c})+\tilde{\rho }(\tilde{c},\tilde{b})$.

$\tilde{\rho }(\tilde{a},\tilde{b})$ is called the fuzzy complex value distance of fuzzy complex numbers $\tilde{a}$ and $\tilde{b}$.

**Remark 9 .**

It can be easily seen that a mapping $\tilde{\rho }:F^{*}(K)\times F^{*}(K)\to F^{*}(K)$ is a fuzzy complex value metric on F*(*K*) if and only if $\tilde{\rho }={\tilde{\rho }}_{1}+i{\tilde{\rho }}_{2}$ for some two fuzzy metrics ${\tilde{\rho }}_{1}$ and ${\tilde{\rho }}_{2}$ on F*(*K*).

**Definition 10 .**

Let $\left\{{\tilde{Z}}_{n}\}\subset F^{*}(K\right)$, $\tilde{Z}\in F^{*}(K)$, and $\tilde{\rho }:F^{*}(K)\times F^{*}(K)\to F^{*}(K)$ be a fuzzy complex value distance, and let $\tilde{\rho }\left({\tilde{Z}}_{n},\tilde{Z}\right)={\tilde{\rho }}_{1}\left({\tilde{Z}}_{n},\tilde{Z}\right)+i{\tilde{\rho }}_{2}\left({\tilde{Z}}_{n},\tilde{Z}\right)$ ($i=\sqrt{-1}$). If for each *ε* > 0, there exists a positive integer *N* such that ${\tilde{\rho }}_{1}\left({\tilde{Z}}_{n},\tilde{Z}\right)<\varepsilon$ and ${\tilde{\rho }}_{2}\left({\tilde{Z}}_{n},\tilde{Z}\right)<\varepsilon$ for all *n* ≥ *N* hold, then $\left\{{\tilde{Z}}_{n}\right\}$ is said to be convergent to $\tilde{Z}$ according to distance $\tilde{\rho }$, denoted by $(\tilde{\rho })\lim_{n\to \infty }{\tilde{Z}}_{n}=\tilde{Z}$.

**Definition 11 .**

Let *Z* be a nonempty complex number set, let *F*(*Z*) be the set of all complex fuzzy sets on *Z*, and let $\tilde{\rho }$ be a fuzzy complex value metric on *F*(*Z*). A complex fuzzy set-value complex fuzzy measure is a mapping $\tilde{\mu }:F(Z)\to F_{+}^{*}(K)$ (where $F_{+}^{*}(K)=\{\tilde{A}+i\tilde{B}|\tilde{A},\tilde{B}\in F_{+}^{*}(R)\}$, $i=\sqrt{-1}$) which satisfies the following conditions:

1. $\tilde{\mu }(⌀)=0$;
2. for any $\tilde{A},\tilde{B}\in F(Z)$ satisfying $\tilde{A}\subset \tilde{B}$, $\tilde{\mu }(\tilde{A})\leq \tilde{\mu }(\tilde{B})$ (i.e., Reμ~(A~)≤ Reμ~(B~) and Im⁡μ~(A~)≤Im⁡μ~(B~));
3. (lower semicontinuous) if $\left\{{\tilde{A}}_{n}\}\subset F(Z\right)$ with $A_{n}\subset {\tilde{A}}_{n+1}$, (*n* = 1,2,…), then $\left(\tilde{\rho })\lim_{n\to \infty }\tilde{\mu }({\tilde{A}}_{n})=\tilde{\mu }({⋃}_{n=1}^{\infty }{\tilde{A}}_{n}\right)$;
4. (upper semicontinuous) if $\{{\tilde{A}}_{n}\}\subset F\left(Z\right)$ with ${\tilde{A}}_{n}⊃{\tilde{A}}_{n+1}$, (*n* = 1,2,…) and $\tilde{\mu }({\tilde{A}}_{n_{0}})\neq \tilde{\infty }$ for some *n*_0_, then $\left(\tilde{\rho })\lim_{n\to \infty }\tilde{\mu }({\tilde{A}}_{n})=\tilde{\mu }({⋂}_{n=1}^{\infty }{\tilde{A}}_{n}\right)$.

Apparently, a complex fuzzy set-value complex fuzzy measure is also a kind of special generalized fuzzy measures.

**Definition 12 .**

A mapping $\tilde{\mu }:F(Z)\to F_{+}^{*}(K)$ is said to be

1. 0-add if $\tilde{\mu }(\tilde{A}\cup \tilde{B})=\tilde{\mu }(\tilde{A})$ for any $\tilde{A},\tilde{B}\in F(Z)$ satisfying $\tilde{A}\cup \tilde{B}\in F(Z)$ and $\tilde{\mu }(\tilde{B})=0$;
2. null-additive (briefly, 0-sub) if $\tilde{\mu }(\tilde{A}\cap {\tilde{B}}^{c})=\tilde{\mu }(\tilde{A})$ for any $\tilde{A},\tilde{B}\in F(Z)$ satisfying $\tilde{A}\cap {\tilde{B}}^{c}\in F(Z)$ and $\tilde{\mu }(\tilde{B})=0$;
3. autocontinuous from above (briefly, autoc. ↓) if $\left(\tilde{\rho }\right)\lim_{n}\tilde{\mu }\left({\tilde{A}}_{n}\cup \tilde{B}\right)=\tilde{\mu }\left(\tilde{B}\right)$ for any for all $\{{\tilde{A}}_{n}\}\subset F\left(Z\right)$ and $\tilde{B}\in F\left(Z\right)$ satisfying ${\tilde{A}}_{n}\cup \tilde{B}\in F\left(Z\right)$, and $\left(\tilde{\rho }\right)\lim_{n}\tilde{\mu }\left({\tilde{A}}_{n}\right)=\tilde{0}$;
4. autocontinuous from below (briefly, autoc. ↑) if $\left(\tilde{\rho }\right)\lim_{n}\tilde{\mu }\left({\tilde{A}}_{n}\cap \tilde{B}\right)=\tilde{\mu }\left(\tilde{B}\right)$ for any for all $\left\{{\tilde{A}}_{n}\}\subset F(Z\right)$ and $\tilde{B}\in F(Z)$ satisfying ${\tilde{A}}_{n}^{c}\cap \tilde{B}\in F(Z)$, and $(\tilde{\rho })\lim_{n}\tilde{\mu }({\tilde{A}}_{n})=\tilde{0}$;
5. autocontinuous if it is both autoc. ↓ and autoc. ↑.

**Definition 13 .**

A complex fuzzy set-value complex fuzzy measure $\tilde{\mu }$ on *F*(*Z*) is said to be pseudo-null-additive (briefly, P.0-add/$\tilde{A}$, where $\tilde{A}\in F(Z)$ with $\tilde{\mu }(\tilde{A})\neq \tilde{\infty }$) if it satisfies $\tilde{\mu }(\tilde{B}\cap \tilde{C})=\tilde{\mu }(\tilde{C})$ for all $\tilde{B}\in F(Z)$ and all $\tilde{C}\in \tilde{A}\cap F(Z)=\{\tilde{A}\cap \tilde{D}|\tilde{D}\in F(Z)\}$ with $\tilde{\mu }(\tilde{A}\cap \tilde{B})=\tilde{\mu }(\tilde{A})$. It is said to be pseudo-null-subtraction (briefly, P.0-sud/$\tilde{A}$, where $\tilde{A}\in F(Z)$ with $\tilde{\mu }(\tilde{A})\neq \tilde{\infty }$) if it satisfies $\tilde{\mu }(\tilde{B}\cap \tilde{C})=\tilde{\mu }(\tilde{C})$ for all $\tilde{B}\in F(Z)$ and all $\tilde{C}\in \tilde{A}\cap F(Z)$ with $\tilde{\mu }(\tilde{A}\cap \tilde{B})=\tilde{\mu }(\tilde{A})$.

**Definition 14 .**

A complex fuzzy set-value complex fuzzy measure $\tilde{\mu }$ on fuzzy *σ*-algebra *F* is said to have property (PGP) if, for each *ε* = *ε*_1_ + *iε*_2_ > 0, there exists a *δ* = *δ*_1_ + *iδ*_2_ > 0 such that $\tilde{\mu }(\tilde{E}\cup \tilde{F})<\varepsilon$ whenever $\tilde{\mu }(\tilde{E})\vee \tilde{\mu }(\tilde{F})<\delta$. It is said to have property (S/A) if, for any $\{{\tilde{B}}_{n}\}\subset F$ with $(\tilde{\rho })\underset{n}{\lim}\tilde{\mu }({\tilde{B}}_{n})=0$, there exists a subsequence $\left\{{\tilde{B}}_{n_{k}}\right\}$ of $\left\{{\tilde{B}}_{n}\right\}$ such that $\tilde{\mu }({⋂}_{j=1}^{\infty }{⋃}_{k=j}^{\infty }{\tilde{B}}_{n_{k}})=0$. It is said to have property (S/B) if $(\tilde{\rho })\lim_{n}\tilde{\mu }({\tilde{B}}_{n})=0$ for any $\{{\tilde{B}}_{n}\}\subset F$.

## 4. Main Results

Let *X* be a set and *F*(*X*) the set of all fuzzy sets on *X*. Then a subfamily *F* ⊂ *F*(*X*) is addable if and only if it satisfies the following conditions (see [21]):

1. *X* ∈ *F*;
2. if $\tilde{A},\tilde{B}\in F$, then $\tilde{A}⊕\tilde{B}$, $\tilde{A}⊝\tilde{B}\in F$,

where $\left(\tilde{A}⊕\tilde{B})(x)=\min(1,\tilde{A}(x)+\tilde{B}(x)\right)$ and $\left(\tilde{A}⊝\tilde{B})(x)=\max(0,\tilde{A}(x)-\tilde{B}(x)\right)$  (for all *x* ∈ *X*).

We first have the following result.

**Theorem 15 .**

Every fuzzy complex measure $\tilde{\mu }$ on a addable class *F* ⊂ *F*(*Z*) is a complex fuzzy value fuzzy complex measure on *F*.

**Proof**

We only prove the upper continuity and lower continuity of $\tilde{\mu }$. Suppose $\left\{{\tilde{A}}_{n}\}\subset F\subset F(Z\right)$, ${\tilde{A}}_{n}↘{⋂}_{n=1}^{\infty }{\tilde{A}}_{n}\in F$, and $\tilde{\mu }({\tilde{A}}_{n_{0}})\neq \tilde{\infty }$ for some *n*_0_. By monotonicity of $\tilde{\mu }$, we have 0≤Reμ~(A~n)≤Reμ~(A~n0) and 0≤Im⁡μ~(A~n)≤Im⁡μ~(A~n0) for any *n* ≥ *n*_0_. Since ${\tilde{A}}_{n_{0}}⊝{\tilde{A}}_{n}↗{\tilde{A}}_{n_{0}}⊝({⋂}_{n=1}^{\infty }{\tilde{A}}_{n})$, we have

(3) Reμ~(A~n0)=(ρ~)lim⁡nReμ~((A~n0⊝A~n)⊕A~n)=(ρ~)lim⁡nReμ~(A~n0⊝A~n)+(ρ~)lim⁡nReμ~(A~n)=Reμ~(A~n0⊝(⋂n=1∞A~n))+(ρ~)lim⁡nReμ~(A~n).

Thereby

(4) Reμ~(A~n0)+Reμ~(⋂n=1∞A~n)  =Reμ~(A~n0⊝(⋂n=1∞A~n))+(ρ~)Reμ~(A~n)   +μ~(⋂n=1∞A~n)  =Reμ~(A~n0)+(ρ~)lim⁡nReμ~(A~n).

It follows that (ρ~)lim⁡nReμ~(A~n)=Reμ~(⋂n=1∞A~n). Similarly, we can prove (ρ~)lim⁡nIm⁡μ~(A~n)=Im⁡μ~(⋂n=1∞A~n), which means that $\tilde{\mu }$ is upper continuous.

Assume $\left\{{\tilde{B}}_{n}\}\subset F\subset F(Z\right)$ and ${\tilde{B}}_{n}↗{⋃}_{n=1}^{\infty }{\tilde{B}}_{n}\in F$. Then $({⋃}_{n=1}^{\infty }{\tilde{B}}_{n})⊝{\tilde{B}}_{n}$ is a monotonic decrease sequence and $({⋃}_{n=1}^{\infty }{\tilde{B}}_{n})⊝{\tilde{B}}_{n}↘⌀$, so

(5) Reμ~(⋃n=1∞B~n)  =Reμ~(((⋃n=1∞B~n)⊝B~n)⊕B~n)  =(ρ~)lim⁡nReμ~((⋃n=1∞B~n)⊝B~n)+(ρ~)lim⁡nReμ~(B~n)  =0+(ρ~)lim⁡nReμ~(B~n)  =(ρ~)lim⁡nReμ~(B~n).

Similarly we can prove Im⁡μ~(⋃n=1∞B~n)=(ρ~)lim⁡nIm⁡μ~(B~n); thus $\tilde{\mu }$ is also lower continuous. In summary, $\tilde{\mu }$ is a complex fuzzy set-value complex fuzzy measure.

**Theorem 16 .**

Every complex fuzzy set-value complex fuzzy measure $\tilde{\mu }$ on a fuzzy *σ*-algebra *F* ⊂ *F*(*Z*) is exhaustive.

**Proof**

Suppose $\{{\tilde{A}}_{n}\}\subset F$ is a disjoin sequence; then

(6) $$\begin{array}{c} \overset{\infty }{\underset{k=n}{⋃}}{\tilde{A}}_{k}↘\overset{\infty }{\underset{n=1}{⋂}}\overset{\infty }{\underset{k=n}{⋃}}{\tilde{A}}_{k}=⌀. \end{array}$$

Assume ${⋂}_{n=1}^{\infty }{⋃}_{k=n}^{\infty }{\tilde{A}}_{k}\neq ⌀$; then Re(∧n=1∞∨k=n∞A~k(z0))>0 for some *z*_0_ ∈ *Z* or Im⁡(∧n=1∞∨k=n∞A~k(z0))>0 for some *z*_0_ ∈ *Z*; that is, Re(∨k=n∞A~k(z0))>0 for all *n* ≥ 1 or Im⁡(∨k=n∞A~k(z0))>0 for all *n* ≥ 1. Without loss of generality, we assume the first. Then there are two distinct indexes *k*_*n*_1__ and *k*_*n*_2__ such that ReA~kn1(z0)>0 and ReA~kn2(z0)>0 (and, thus, Re(A~kn1(z0)∧A~kn2(z0))>0), which conflicts with the fact that $\left\{{\tilde{A}}_{n}\right\}$ is a disjoin sequence. Therefore ${⋃}_{k=n}^{\infty }{\tilde{A}}_{k}↘{⋂}_{n=1}^{\infty }{⋃}_{k=n}^{\infty }{\tilde{A}}_{k}=⌀$ holds. Thus

(7) (ρ~)lim⁡nReμ~(A~n)≤(ρ~)lim⁡nReμ~(⋃k=n∞A~k)=Re(⌀)=0,(ρ~)lim⁡nIm⁡μ~(A~n)≤(ρ~)lim⁡nIm⁡μ~(⋃k=n∞A~k)  =Im⁡(⌀)=0.

Then we get $(\tilde{\rho })\lim_{n}\tilde{\mu }({\tilde{A}}_{n})\leq 0$.

On the other hand, since $\tilde{\mu }$ is complex fuzzy set-value complex fuzzy measure on the fuzzy *σ*-algebra *F*, $(\tilde{\rho })\lim_{n}\tilde{\mu }({\tilde{A}}_{n})\geq 0$, so $(\tilde{\rho })\lim_{n}\tilde{\mu }({\tilde{A}}_{n})=0$.

**Theorem 17 .**

If $\tilde{\mu }\left(F\right)=\{\tilde{\mu }(\tilde{A})|\tilde{A}\in F\}\in A^{*}$, then

(8) Reμ~(lim⁡_n→∞A~n)≤(ρ~)lim⁡_n→∞Reμ~(A~n)≤(ρ~)lim⁡¯n→∞Reμ~(A~n)≤Reμ~(lim⁡¯n→∞A~n),Im⁡μ~(lim⁡_n→∞A~n)≤(ρ~)lim⁡_n→∞Im⁡μ~(A~n)≤(ρ~)lim⁡¯n→∞Im⁡μ~(A~n)≤Im⁡μ~(lim⁡¯n→∞A~n).

**Proof**

Since ${⋂}_{n=k}^{\infty }{\tilde{A}}_{n}↗$ about *k*, Reμ~(⋃k=1∞⋂n=k∞A~n)= (ρ~)lim⁡nReμ~(⋂n=k∞A~n) and Im⁡μ~(⋃k=1∞⋂n=k∞A~n)=(ρ~)lim⁡nIm⁡μ~(⋂n=k∞A~n).

As Reμ~(⋂n=k∞A~n)≤Reμ~(A~n) and Im⁡μ~(⋂n=k∞A~n)≤Im⁡μ~(A~n) for all *n* ≥ *k*, Reμ~(⋂n=k∞A~n)≤inf⁡n≥kReμ~(A~n) and Im⁡μ~(⋂n=k∞A~n)≤inf⁡n≥kIm⁡μ~(A~n). Therefore

(9) Reμ~(lim⁡_n→∞A~n)=Reμ~(⋃k=1∞⋂n=k∞A~n)≤(ρ~)lim⁡k→∞inf⁡n≥kReμ~(A~n)=(ρ~)lim⁡_n→∞Reμ~(A~n),Im⁡μ~(lim⁡_n→∞A~n)=Im⁡μ~(⋃k=1∞⋂n=k∞A~n)≤(ρ~)lim⁡k→∞inf⁡n≥kIm⁡μ~(A~n)=(ρ~)lim⁡_n→∞Im⁡μ~(A~n),

which means Reμ~(lim⁡_n→∞A~n)≤(ρ~)lim⁡_n→∞Reμ~(A~n).

Similarly, Im⁡μ~(lim⁡_n→∞A~n)≤(ρ~)lim⁡_n→∞Im⁡μ~(A~n).

From properties of upper limits and lower limits we can see the following theorem holds.

**Theorem 18 .**

Let $\tilde{\mu }:F(Z)\to F_{+}^{*}(K)=\{\tilde{A}+i\tilde{B}:\tilde{A},\tilde{B}\in F_{+}^{*}(R),i=\sqrt{-1}\}$ and $\tilde{E}\in F(Z)$. If $\tilde{\mu }$ is 0-addable and upper continuous on *F*(*Z*), then, for any $\{{\tilde{A}}_{n}\}\subset F\left(Z\right)$ satisfying ${\tilde{A}}_{n}⊃{\tilde{A}}_{n+1}\;\;\left(n=1,2,\ldots \right)$, $(\tilde{\rho })\lim_{n}\tilde{\mu }({\tilde{A}}_{n})=\tilde{0}$ and $\tilde{\mu }(\tilde{E}\cup {\tilde{A}}_{n_{0}})\neq \tilde{\infty }$, $\left(\tilde{\rho })\lim_{n}\tilde{\mu }(\tilde{E}\cup {\tilde{A}}_{n})=\tilde{\mu }(\tilde{E}\right)$.

**Proof**

Let $\tilde{A}={⋂}_{n=1}^{\infty }{\tilde{A}}_{n}$. Since $\tilde{\mu }$ is upper continuous,

(10) Reμ~(A~)=Reμ~(⋂n=1∞A~n)=(ρ~)lim⁡nReμ~(A~n)=0~,Im⁡μ~(A~)=Im⁡μ~(⋂n=1∞A~n)=(ρ~)lim⁡nIm⁡μ~(A~n)=0~.

As $\tilde{E}\cup {\tilde{A}}_{n}↘\tilde{E}\cup \tilde{A}$ and $\tilde{\mu }$ is upper continuous and 0-addable, we have

(11) (ρ~)lim⁡nReμ~(E~∪A~n)=Reμ~(E~∪A~)=Reμ~(E~),(ρ~)lim⁡nIm⁡μ~(E~∪A~n)=Im⁡μ~(E~∪A~)=Im⁡μ~(E~).

So $\left(\tilde{\rho })\lim_{n}\tilde{\mu }(\tilde{E}\cup {\tilde{A}}_{n})=\tilde{\mu }(\tilde{E}\right)$ holds.

Similar to Theorem 18, we have the following.

**Theorem 19 .**

Let $\tilde{\mu }:F(Z)\to F_{+}^{*}(K)=\{\tilde{A}+i\tilde{B}:\tilde{A},\tilde{B}\in F_{+}^{*}(R),i=\sqrt{-1}\}$ and $\tilde{E}\in F(Z)$. If $\tilde{\mu }$ is 0-subtractable and continuous on *F*(*Z*), then, for any $\{{\tilde{E}}_{n}\}\subset F\left(Z\right)$ satisfying ${\tilde{E}}_{n}⊃{\tilde{E}}_{n+1}\;\;\left(n=1,2,\ldots \right)$ and $\left(\tilde{\rho })\lim_{n}\tilde{\mu }({\tilde{E}}_{n})=\tilde{0},\;\;(\tilde{\rho })\lim_{n}\tilde{\mu }(\tilde{E}\cap {{\tilde{E}}^{c}}_{n})=\tilde{\mu }(\tilde{E}\right)$.

**Theorem 20 .**

Assume that $\tilde{A}\in F\subset F(Z)$, $\tilde{\mu }$ is a complex fuzzy set-value complex fuzzy measure on *F*(*Z*) which is pseudo-zero addable about $\tilde{A}$, and $\tilde{\mu }(\tilde{A})\neq \tilde{\infty }$. If $\left(\tilde{\rho })\lim_{n}\tilde{\mu }(\tilde{A}\cap {\tilde{B}}_{n})=\tilde{\mu }(\tilde{A}\right)$ for any $\{{\tilde{B}}_{n}\}\subset F$ satisfying ${\tilde{B}}_{n}↑$, then $\left(\tilde{\rho })\lim_{n}\tilde{\mu }(\tilde{C}\cup (\tilde{A}\cap {{\tilde{B}}^{c}}_{n}))=\tilde{\mu }(\tilde{C}\right)$ for any $\tilde{C}\in \tilde{A}\cap F$.

**Proof**

Let $\tilde{B}={⋃}_{n=1}^{\infty }{\tilde{B}}_{n}$. As $\tilde{\mu }$ is lower continuous, we have

(12) Reμ~(A~∩B~)=Reμ~(A~∩⋃n=1∞B~n)=Reμ~(⋃n=1∞(A~∩B~n))=(ρ~)lim⁡nRe(A~∩B~n),Im⁡μ~(A~∩B~)=Im⁡μ~(A~∩⋃n=1∞B~n)=Im⁡μ~(⋃n=1∞(A~∩B~n))=(ρ~)lim⁡nIm⁡(A~∩B~n).

Therefore $\tilde{\mu }\left(\tilde{A}\cap \tilde{B}\right)=\tilde{\mu }\left(\tilde{A}\right)$. As $\tilde{A}\cap {\tilde{B}}_{n}^{c}↘\tilde{A}\cap {\tilde{B}}^{c}$, $\tilde{C}\cup \left(\tilde{A}\cap {\tilde{B}}_{n}^{c}\right)↘\tilde{C}\cup \left(\tilde{A}\cap {\tilde{B}}^{c}\right)$ for any $\tilde{C}\in \tilde{A}\cap F$. By upper continuity and P.0-add$/\tilde{A}$ of $\tilde{\mu }$, we have

(13) (ρ~)lim⁡nReμ~(C~∪(A~∩B~nc))  =Reμ~(⋂n=1∞(C~∪(A~∩B~nc)))  =Reμ~(C~∪(A~∩B~c))=Reμ~(C~),(ρ~)lim⁡nIm⁡μ~(C~∪(A~∩B~nc))  =Im⁡μ~(⋂n=1∞(C~∪(A~∩B~nc)))  =Im⁡μ~(C~∪(A~∩B~c))=Im⁡μ~(C~).

Hence $\left(\tilde{\rho })\lim_{n}\tilde{\mu }(\tilde{C}\cup (\tilde{A}\cap {{\tilde{B}}^{c}}_{n}))=\tilde{\mu }(\tilde{C}\right)$.

Similarly, we have the following.

**Theorem 21 .**

Suppose that $\tilde{A}\in F\subset F\left(Z\right)$, $\tilde{\mu }$ is a complex fuzzy set-value complex fuzzy measure on *F*(*Z*) which is pseudo-zero subtractable about $\tilde{A}$, and $\tilde{\mu }(\tilde{A})\neq \tilde{\infty }$. If $\left(\tilde{\rho })\lim_{n}\tilde{\mu }(\tilde{A}\cap {\tilde{B}}_{n})=\tilde{\mu }(\tilde{A}\right)$ for any $\{{\tilde{B}}_{n}\}\subset F$ satisfying ${\tilde{B}}_{n}↑$, then $\left(\tilde{\rho })\lim_{n}\tilde{\mu }(\tilde{C}\cap {\tilde{B}}_{n})=\tilde{\mu }(\tilde{C}\right)$ for any $\tilde{C}\in \tilde{A}\cap F$.

**Theorem 22 .**

Let $\tilde{\mu }$ be a complex fuzzy set-value complex fuzzy measure on a fuzzy *σ*-algebra *F* ⊂ *F*(*Z*) and $\tilde{\mu }\left(F\right)=\{\tilde{\mu }\left(\tilde{A}\right):\tilde{A}\in F\}\in A^{*}$. If $\tilde{\mu }$ is *autoc*. ↓, then $\tilde{\mu }$ possesses (PGP) property.

**Proof**

Suppose that $\tilde{\mu }$ does not possess (P.G.P) property; then there exists an *ε*_0_ = *ε*_0_′ + *iε*_0_′′ (where *ε*_0_′, *ε*_0_′′ are positive real numbers) such that, for any natural numbers *n*, *m*, there exist $\{{\tilde{E}}_{n}\},\{{\tilde{F}}_{n}\}\subset F$ such that

(14) Reμ~(E~n)∨Reμ~(F~n)<1n,Im⁡μ~(E~n)∨Im⁡μ~(F~n)<1m,Reμ~(E~n∪F~n)≮ε0′,  Im⁡μ~(E~n∪F~n)≮ε0′′.

Thus $\tilde{\mu }({\tilde{E}}_{n}\cup {\tilde{F}}_{n})≮\varepsilon_{0}$ and, thus, (ρ~)lim⁡nReμ~(E~n)=(ρ~)lim⁡nReμ~(F~n)=0 and (ρ~)lim⁡nIm⁡μ~(E~n)=(ρ~)lim⁡nIm⁡μ~(F~n)=0. From upper autocontinuity of $\tilde{\mu }$, we have

(15) (ρ~)lim⁡nReμ~(E~n∪F~n)=0,  (ρ~)lim⁡nIm⁡μ~(E~n∪F~n)=0.

Therefore $\tilde{\mu }({\tilde{E}}_{n}\cup {\tilde{F}}_{n})<\varepsilon_{0}$ for some *n*_0_ ≥ 1. This conflicts with the hypothesis.

**Theorem 23 .**

Suppose that $\tilde{\mu }$ possesses (P.G.P) property. If $\{{\tilde{E}}_{n}\}\subset F$ and $(\tilde{\rho })\lim_{n}\tilde{\mu }({\tilde{E}}_{n})=0$, then there exists a sequence {*δ*_*n*_} of real numbers satisfying *δ*_*n*_ > 0 (for all n) and *δ*_*n*_↘0 and a subsequence $\left\{{\tilde{E}}_{n_{k}}\right\}$ of $\left\{{\tilde{E}}_{n}\right\}$ such that $\tilde{\mu }({⋃}_{i=k+1}^{\infty }{\tilde{E}}_{n_{i}})<\delta_{k}$ (for all *k* ≥ 1). Furthermore, $\tilde{\mu }$ possesses (SA) property.

**Proof**

For any real numbers *ε*′, *ε*′′, *δ*_1_′, *δ*_1_′′ > 0, let *ε* = *ε*′ + *iε*′′, *δ*_1_ = *δ*_1_′ + *iδ*_1_′′, *δ*_1_′ ∈ (0, *ε*′), and *δ*_1_′′ ∈ (0, *ε*′). Since $\tilde{\mu }$ possesses (P.G.P) property, there exists a *δ*_1_ ∈ (0, *ε*) such that Reμ~(E~∪F~)<ε′ and Im⁡μ~(E~∪F~)<ε′ whenever Reμ~(E~)∨μ~(F~)<δ1′ and Im⁡μ~(E~)∨μ~(F~)<δ1′′. Since $\left(\tilde{\rho }\right)\lim_{n}\tilde{\mu }\left({\tilde{E}}_{n}\right)=0$, there exists an *n*_1_ such that Reμ~(E~n1∪F~)<ε′, Im⁡μ~(E~n1∪F~)<ε′′ whenever Reμ~(E~n1)<δ1′ and Im⁡μ~(E~n1)<δ1′′. Therefore $\tilde{\mu }\left({\tilde{E}}_{n_{1}}\cup \tilde{F}\right)<\varepsilon$. Since $\tilde{\mu }$ possesses (P.G.P) property, there exists a *δ*_2_ = *δ*_2_′ + *iδ*_2_′′ such that *δ*_2_′ ∈ (0, *δ*_1_′∧*ε*′/2), *δ*_2_′′ ∈ (0, *δ*_1_′′∧*ε*′′/2), Reμ~(E~∪F~)<δ1′, and Im⁡μ~(E~∪F~)<δ2′′ whenever Re(μ~(E~)∨μ~(F~))<δ2′ and Im⁡(μ~(E~)∨μ~(F~))<δ2′′. As *δ*_2_ = *δ*_2_′ + *iδ*_2_′′ > 0, there exists an *n*_2_ > *n*_1_ such that Reμ~(E~n2)<δ2′ and Im⁡μ~(E~n2)<δ2′′. Hence Reμ~(E~n2∪F~)<δ1′, Im⁡μ~(E~n2∪F~)<δ2′′, and, thus, Reμ~(E~n1∪E~n2∪F~)<ε′ and Im⁡μ~(E~n1∪E~n2∪F~)<ε′′.

For *δ*_2_ = *δ*_2_′ + *iδ*_2_′′ > 0 and there exists *δ*_3_ = *δ*_3_′ + *iδ*_3_′′ > 0, *δ*_3_′ ∈ (0, *δ*_2_′∧*ε*/2^2^), and *δ*_3_′′ ∈ (0, *δ*_2_′′∧*ε*/2^2^) such that Re(μ~(E~)∨μ~(F~))<δ3′, Im⁡(μ~(E~)∨μ~(F~))<δ3′′⇒

(16) Reμ~(E~∪F~)<δ2′,  Im⁡μ~(E~∪F~)<δ2′′.

For *δ*_3_ = *δ*_3_′ + *iδ*_3_′′ > 0, *δ*_3_′ ∈ (0, *δ*_2_′∧*ε*/2^2^), *δ*_3_′′ ∈ (0, *δ*_2_′′∧*ε*/2^2^) since $(\tilde{\rho })\lim_{n}\tilde{\mu }({\tilde{E}}_{n})=0$, there exists *n*_3_ > *n*_2_, such that Reμ~(E~n3)<δ3′ and Im⁡μ~(E~n3)<δ3′′. Therefore $\tilde{\mu }\left({\tilde{E}}_{n_{3}}\cup \tilde{F}\right)<\delta_{2}$, $\tilde{\mu }\left({\tilde{E}}_{n_{3}}\cup {\tilde{E}}_{n_{2}}\cup \tilde{F}\right)<\delta_{1}$, and $\tilde{\mu }\left({\tilde{E}}_{n_{3}}\cup {\tilde{E}}_{n_{2}}\cup {\tilde{E}}_{n_{1}}\cup \tilde{F}\right)<\varepsilon$.

Generally we can get *n*_*k*+1_ > *n*_*k*_ > *n*_*k*−1_ > ⋯>*n*_1_, and *δ*_*k*_ < *δ*_*k*−1_∧*ε*/2^*k*−1^, such that $\tilde{\mu }({⋃}_{i=k}^{r+1}{\tilde{E}}_{n_{i}})<\delta_{k-1}$, (*k* = 1,2, 3,…, *r* + 1, *r* ≥ 1).

Let ${\tilde{B}}_{k}={⋃}_{i=k}^{\infty }{\tilde{E}}_{n_{i}}$and $\tilde{E}={⋂}_{k=2}^{\infty }{⋃}_{i=k}^{\infty }{\tilde{E}}_{n_{i}}={⋂}_{k=1}^{\infty }{\tilde{B}}_{k}$; then ${\tilde{B}}_{k}↘\tilde{E}$, $\tilde{\mu }\left({\tilde{B}}_{k}\right)=\tilde{\mu }\left({⋃}_{i=k}^{\infty }{\tilde{E}}_{n_{i}}\right)\leq \delta_{k-1},\left(k\geq 1\right)$.

Hence $\tilde{\mu }(\tilde{E})=0$; that is, $\tilde{\mu }$ possesses (SA) property.
